# Psychological distress and workplace psychosocial risks

**DOI:** 10.1192/j.eurpsy.2026.11818

**Published:** 2026-07-31

**Authors:** I. Sellami, A. Abbes, A. Feki, M. L. Masmoudi, M. Hajjaji, K. Jmal Hammami

**Affiliations:** 1 occupational medicine; 2 Rheumatoloy, Hedi Chaker Hospital, sfax, Tunisia

## Abstract

**Introduction:**

Concern is increasing regarding the relationship between psychological distress and psychosocial risks in the workplace. Both psychological distress and psychosocial risks are significant challenges to workers’ health, well-being, and productivity.

**Objectives:**

This study aimed to investigate the relationship between psychological distress and psychosocial risks within the workplace settings.

**Methods:**

The study was carried out among a sample of workers. Data collection took place from January to June 2022 through a self-administered survey, which included items on socio-professional characteristics, the Kessler Psychological Distress Scale, and the validated French version of the Karasek questionnaire.

**Results:**

This study included 70 workers. The mean age was 38.1 ±10.2 years. The mean of the job tenure was 14.5±11.1 years. About half of the electricians had high psychological demand (47.1%), 61.4% had a low latitude, and 70% had low social support. The Karasek model indicated that 31.4% of employees were in a state of job strain, with 25.4% facing iso-strain. We found that 10% of participants presented a probability of a serious mental illness. Psychological distress was associated with high psychological demand and low social support.

**Conclusions:**

Findings from this study indicate that psychological distress is closely linked with psychosocial risks in the workplace, and both are common among workers. These factors represent a major concern for occupational health systems. Promoting mental health effectively requires taking into account the particular work conditions that increase exposure to psychosocial risks.

**Disclosure of Interest:**

None Declared

